# In-vivo lung fibrosis staging in a bleomycin-mouse model: a new micro-CT guided densitometric approach

**DOI:** 10.1038/s41598-020-71293-3

**Published:** 2020-10-30

**Authors:** Laura Mecozzi, Martina Mambrini, Francesca Ruscitti, Erica Ferrini, Roberta Ciccimarra, Francesca Ravanetti, Nicola Sverzellati, Mario Silva, Livia Ruffini, Sasha Belenkov, Maurizio Civelli, Gino Villetti, Fabio Franco Stellari

**Affiliations:** 1 Department of Medicine and Surgery, University of Parma, Parma, Italy; 2 Department of Veterinary Science, University of Parma, Parma, Italy; 3 Phamacology and Toxicology Department, Chiesi Farmaceutici S.P.A., Corporate Pre-Clinical R&D, Largo Belloli, 11/A 43122, Parma, Italy; 4 Department Nuclear Medicine, Academic Hospital of Parma, Parma, Italy; 5 Perkin Elmer, Inc., Waltham, MA USA

**Keywords:** 3-D reconstruction, X-ray tomography, Imaging, Biological models, Animal disease models, Respiratory system models, Pharmaceutics, Biological techniques, Drug discovery

## Abstract

Although increasing used in the preclinical testing of new anti-fibrotic drugs, a thorough validation of micro-computed tomography (CT) in pulmonary fibrosis models has not been performed. Moreover, no attempts have been made so far to define density thresholds to discriminate between aeration levels in lung parenchyma. In the present study, a histogram-based analysis was performed in a mouse model of bleomycin (BLM)-induced pulmonary fibrosis by micro-CT, evaluating longitudinal density changes from 7 to 21 days after BLM challenge, a period representing the progression of fibrosis. Two discriminative densitometric indices (i.e. 40th and 70th percentiles) were extracted from Hounsfield Unit density distributions and selected for lung fibrosis staging. The strong correlation with histological findings (r_Spearman_ = 0.76, p < 0.01) confirmed that variations in 70th percentile could reflect a pathological lung condition and estimate the effect of antifibrotic treatments. This index was therefore used to define lung aeration levels in mice distinguishing in hyper-inflated, normo-, hypo- and non-aerated pulmonary compartments. A retrospective analysis performed on a large cohort of mice confirmed the correlation between the proposed preclinical density thresholds and the histological outcomes (r_Spearman_ = 0.6, p < 0.01), strengthening their suitability for tracking disease progression and evaluating antifibrotic drug candidates.

## Introduction

Computed tomography (CT) imaging is now an invaluable tool for both qualitative and quantitative assessment of numerous lung disorders in clinical practice, as confirmed by the international diagnostic guidelines^1–3^. Indeed, it is increasingly recognized that human observation has poor reproducibility in the quantification of the extent of diffuse lung disease^4^. Owing to the linear relationship between X-ray attenuation and tissue density, lung densitometry has been shown to be superior to visual assessment in several disorders: it is widely available, reproducible and much less time consuming than visual scoring^5^. Histogram-based measurements refer to CT numbers frequency distribution (i.e. physical density distribution). Hence, information on aeration levels in specific lung regions (compartments) can be derived from lung density histograms^5–9^. Variation in lung densitometry has been used as an endpoint of studies testing drug efficacy in the treatment of several lung pathologies^4,10,11^. For instance, CT densitometry has been shown to be accurate in the assessment of emphysema-modifying therapy^12^.

Micro-CT based quantitative tools have been used in several animal models to understand the pathogenesis of lung diseases, resulting in accurate longitudinal assessment of disease progression and compliance with the 3R rules (Refinement, Replacement, Reduction)^13–17^. Unfortunately, despite the strength and potential of this technology in preclinical research, there are still scarce data on CT number (i.e. Hounsfield Units-HU) distributions and no clear guidelines for animal lungs^17–19^. In contrast to the large number of reports for human lungs^12,20–22^, no cut-off densitometric values have been proposed so far for both identifying and quantifying lung abnormalities such as fibrosis and emphysema in animal models. To the best of our knowledge, the HU ranges used to define differently aerated lung compartments vary from investigator to investigator, depending on the animal species or on the disease (Table1)^17,19,21,23–25^**.** Nevertheless, standardized density thresholds for characterizing lung disorders could be of critical importance when comparing different animal models or testing different treatments in drug discovery processes^18^. In this study, we used a murine model of lung fibrosis [induced by bleomycin (BLM) administration]^25–28^ to provide a complete mapping of lung density and quantitative densitometric metrics for the evaluation of disease progression and antifibrotic effect.

**Table 1:** Hounsfield units (HU) ranges used to define aeration levels in lung tissue, for animal models describing different pathologies.

Authors	Disease	Low density	Normal density	High density	Species
Johnson, 2007^23^	Fibrosis	X	[− 1,000, − 500] HU	[− 500, − 100] HU	Rats
Reske, 2011^24^	X	[− 1,000, − 900] HU	[− 900, − 500] HU	[− 100, + 100] HU	Pigs/sheep
Saito, 2012^17^	Radiation induced injury	< − 500 HU	X	[− 500, − 200] HU	Mice
		Peak HU: peak of − 200 HU to − 800 HU			
		Number- 1,000: # px at − 1,000 HU			
De Langhe, 2012^25^	Fibrosis and emphysema	Air; containing px< − 383 HU			Mice
Perez, 2017^19^	Radiation induced injury	X	[− 900, − 400] HU	Fibrotic: [− 200, + 200] HU	Rats
Bell, 2018^21^	RA-ILD	Peak HU defining aerated and non-aerated: − 256 HU			Mice

*IPF* idiopathic pulmonary fibrosis, *RA-ILD* rheumatoid arthritis associated interstitial lung disease.

## Methods

A total of 300 mice underwent imaging and image processing procedures, as detailed below, in 11 drug discovery studies conducted between 2017 and 2019. Since a drug efficacy study was not the purpose of the analysis, no mention of the specific compounds is made, except for Nintedanib (60 mg/kg/day by oral gavage), an FDA-approved drug with demonstrated antifibrotic efficacy in the BLM mouse model^29^.

### Experimental animals

Female inbred C57Bl/6 (7- to 8-week old) mice were purchased from Envigo, Italy (San Pietro al Natisone, Udine, Italy). Prior to use, animals were acclimatized for at least 5 days to the local vivarium conditions (room temperature: 20–24 °C; relative humidity: 40–70%; 12-h light–dark cycle), having free access to standard rodent chow and softened tap water. All animal experiments described herein were approved by the intramural animal-welfare committee for animal experimentation of Chiesi Farmaceutici under protocol 449/2016-PR, and in compliance with European Directive 2010/63 UE, Italian D.Lgs 26/2014 and the revised “Guide for the Care and Use of Laboratory Animals”^30^.

### Bleomycin administration

Animals were lightly anesthetized with 2.5% isoflurane delivered in a box and BLM hydrochloride [BAXTER (1 mg/kg) in 50 µl saline (0.9%) or vehicle (50 µl saline (0.9%)] was administered via oropharyngeal aspiration (OA) using a micropipette^31^. Mice were positioned on the intubation platform, hanging them by their incisors placed on the wire. The tongue was pulled out and held with forceps, the liquid was placed onto the distal part of the oropharynx with a micropipette and the nose was gently closed until the liquid disappeared. Mice were monitored in cages until they had fully recovered. This procedure was performed on day 0 and 4 (25 μg/mouse for each instillation). On day 7, mice were divided into three groups: healthy saline (vehicle), pathological (BLM) and drug-treated. Each treatment used in this work (different unspecified compounds) was started on day 7 (i.e. baseline), when fibrosis was well established, and lasted continued to day 21, when extensive fibrotic lesions were still evident. All the mice were orally treated daily for 2 weeks, either with the vehicle or the drug under investigation. The OA protocol was shown to give a uniform distribution of fibrotic lesions through the lung, allowing an easy detection of parenchymal changes^32^. All mice were weighed daily from the beginning of the trial.

### Histology

After sacrifice, the lungs were harvested. Lungs were removed and inflated with a cannula through the trachea by gentle infusion of 0.6 ml of 10% neutral-buffered formalin and fixed for 24 h. For histological assessment, the samples were dehydrated in a graded ethanol series, clarified in xylene and embedded in paraffin. Sections of 5 μm thickness were cut with a rotary microtome (Slee Cut 6062, Slee Medical, Mainz, Germany). The sections were stained with Hematoxylin and Eosin (H&E) and Masson’s trichrome (TM), according to the manufacturer’s specifications (Histo-Line Laboratories). The whole-slide images (WSI) were acquired by the NanoZoomer S-60 Digital slide scanner (Hamamatsu, Japan) for analysis. Two independent researchers, with experience in animal models of lung fibrosis, performed blinded histological analyses of the specimens/slides. Fibrotic modifications were assessed morphologically and semi-quantitatively graded according to the scale defined by Ashcroft et al.^33^ and modified by Hübner et al.^34^. Three sections for each lung sample were stained with Masson’s Trichrome, and scored on a scale of 0–8. The final score was expressed as a mean of individual scores observed across all microscopic fields. In order to quantify the distribution of pulmonary fibrosis, the Ashcroft scores were graded in 3 classes of increasing values: ranging from 0–3 (mild), 4 (moderate) and ≥ 5 (severe)^35^.

### Micro-computed tomography acquisition protocol

Following anesthesia induction and maintenance with 2% isoflurane, mice lungs were scanned with a Quantum GX Micro-CT (PerkinElmer, Inc. Waltham, MA) at 21 days. For time-course studies, lung imaging was also performed at day 7. The microfocus X-ray source in this scanner uses a Tungsten anode. A fixed filter of 0.5 mm Aluminium (Al) and 0.06 mm Copper (Cu) is placed in front of the exit port to remove low energy X-rays that contribute to dose but do not improve image quality. Images were acquired with a respiratory gated technique with the following parameters: X-ray tube voltage 90 KV, X-ray tube current 88 µA, total scan time of 4 min. A ring reduction correction was applied to the sinograms and the entire set of projection radiographs was input into a GPU-based filtered back-projection algorithm with a Ram-Lak filter^36^. The retrospectively gated acquisition protocol in ‘high speed’ mode (acquiring projections without averaging in list-mode over a total angle of 360°), resulted in two 3D datasets with 50 μm isotropic reconstructed voxel size, corresponding to the two different phases of the breathing cycle (i.e. inspiration and expiration). Data reported here refer to the end of expiration phase. The system is calibrated monthly with standard phantoms for noise, uniformity, low contrast and resolution (Micro-CT phantoms, Quality Assurance in Radiology and Medicine, Germany).

### Image post-processing: lung segmentation protocols and analysis

**Figure 1:**
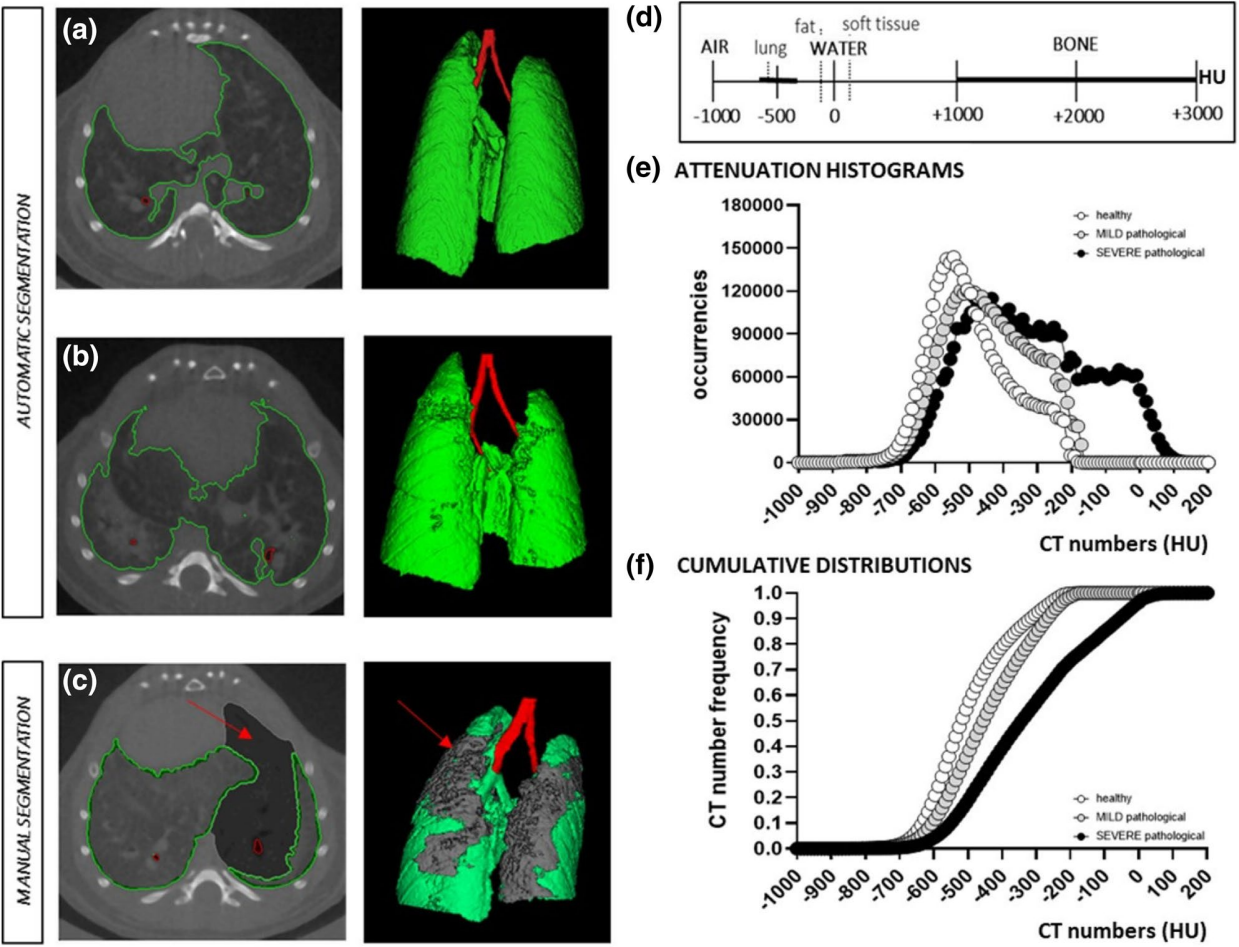
Lung segmentation protocols and analysis**. (a, b)** Semi-automatic segmentation for the extraction of lung parenchyma (green) and airways (red) in healthy subjects **(a)** or in mild lesioned pathological subjects **(b)**; **(c)** manual segmentation applied in presence of severely damaged parenchyma. The undetectable volume is highlighted in grey. **(d)** The Hounsfield scale of CT numbers (HU). **(e, f)** Examples of HU density histograms **(e)** obtained from lung segmentation maps in vehicles (white) and BLM with mild and severe histological lesions (in grey and black, respectively), at 21 days. In **(f)** the cumulative frequency histograms for the same subjects. Analyze 12.0 (Mayo Clinic, Rochester, MN) was used for μCT data analyses, https://www.analyzedirect.com. Figures were created using GraphPad Prism 8 (GraphPad Software, La Jolla, CA, USA), https://www.graphpad.com.

For each acquisition, a stack of 512 cross-sectional images stored in unsigned 16-bit file format was produced. The reconstructed datasets were imported and analyzed using Analyze software (Analyze 12.0; Copyright 1986–2017, Biomedical Imaging Resource, Mayo Clinic, Rochester, MN). A 5 × 5 × 5 kernel size median filter was always applied to image stacks. A conversion scale was used to express grey levels as CT numbers (Hounsfield Units—HU), setting -1,000 HU as the density of air and 0 HU as the density of water Fig. 1(d).

Pulmonary image analysis is based on the creation of a 3D segmentation map in which two main objects of interest must be included: airways and lungs.

A semi-automatic segmentation is always used to define airways: an object extractor tool enables the specification of a seed point and a threshold range to properly detect the object in the volume. The accurate and precise segmentation of lungs boundaries is a critical step and it is essential for the extraction of densitometric indices^5^. In drug screening, two basic approaches are usually employed to create the lung segmentation map (Fig. 1), depending on the aeration level of parenchyma (see Supplementary Video 1 and Supplementary Video 2):

- A semi-automatic segmentation is used to define the whole lung volume when the parenchyma is detectable and well defined by the threshold range (e.g. vehicles or mild and moderate lesions in pathological subjects) Fig. 1a, b**.** This approach was considered acceptable for the BLM-induced murine fibrosis model analyzed herein and it was used to define aeration levels in mice as detailed below.
- A manual segmentation is necessary to correctly identify lung regions that are not clearly detectable due to lack of aeration, absence of clear boundaries and jagged or severely damaged parenchyma (i.e. areas of massive fibrosis in pathological subjects)^35^ (Fig. 1c)**.** The manual approach was required for only 2–3% of the mice that underwent the OA administration procedures described previously. In these cases, an alternative protocol based on a mathematical procedure was developed and proposed to avoid manual intervention when a large number of animals needs to be screened (see predictive lung volume method in the following section).

To characterize global changes in the lung during fibrosis progression and to evaluate the therapeutic effects of the compounds under investigation, we used the segmentation map to extract several micro-CT quantitative parameters: lung volume [mm^3^], mean lung density [HU], standard deviation of the mean, maximum and minimum HU values within the region-of-interest (ROI). In addition, the attenuation histograms were generated from lung ROIs using bins of 10HU width (range HU: [− 1,000, 0]; 100 bins), and the corresponding cumulative distributions were derived for each time point, as reported in Fig. 1e, f respectively.

Longitudinal HU changes, between the beginning and the end of the pharmacological treatment (7 and 21 days), were used to identify a measurable read-out from lung density histograms^20^. To test whether the derived quantitative densitometric indices were able to discriminate parenchymal changes between healthy, pathological and drug-treated mice, their mean HU values for each group were extracted from cumulative distributions and compared.

### Predictive lung volume method: an approximation rule

In fibrosis drug discovery we need to deal with several animal models, each experiment involving a large number of animals. Different degrees of fibrotic lesions can be revealed: mainly mild or moderate (as observed for the BLM model described herein) or more severe, as found for 2–3% of the analyzed animals. Denser and tissue-like regions, comparable to soft tissues such as thoracic wall, mediastinum or diaphragm, are closely associated with severe lung fibrotic damage. The HU distributions for animals with severe pathology (i.e. black curve in Fig. 1e) could include the fibrotic volume, showing HU values close to zero and requiring a manual segmentation. With the aim of reducing both the computation times and operator-dependent variability that are inherent to this approach, we defined a method to estimate total lung volumes, even with lesioned lungs for which the automatic segmentation is not able to give a reliable output. The approximation rule obtained, called *`predictive lung volume method`,* can be used to avoid manual segmentation procedures with acceptable risk of bias.

Pathological animals from BLM groups characterized by different levels of fibrotic lesions were manually segmented to extract lung ROIs and volumes, including (if any) the denser portions (n = 40). These groups consisted of fibrotic animals from both the above described OA model (2–3% of the mice) and an intratracheal administration-based model, formerly investigated by the group^28^.

**Figure 2:**
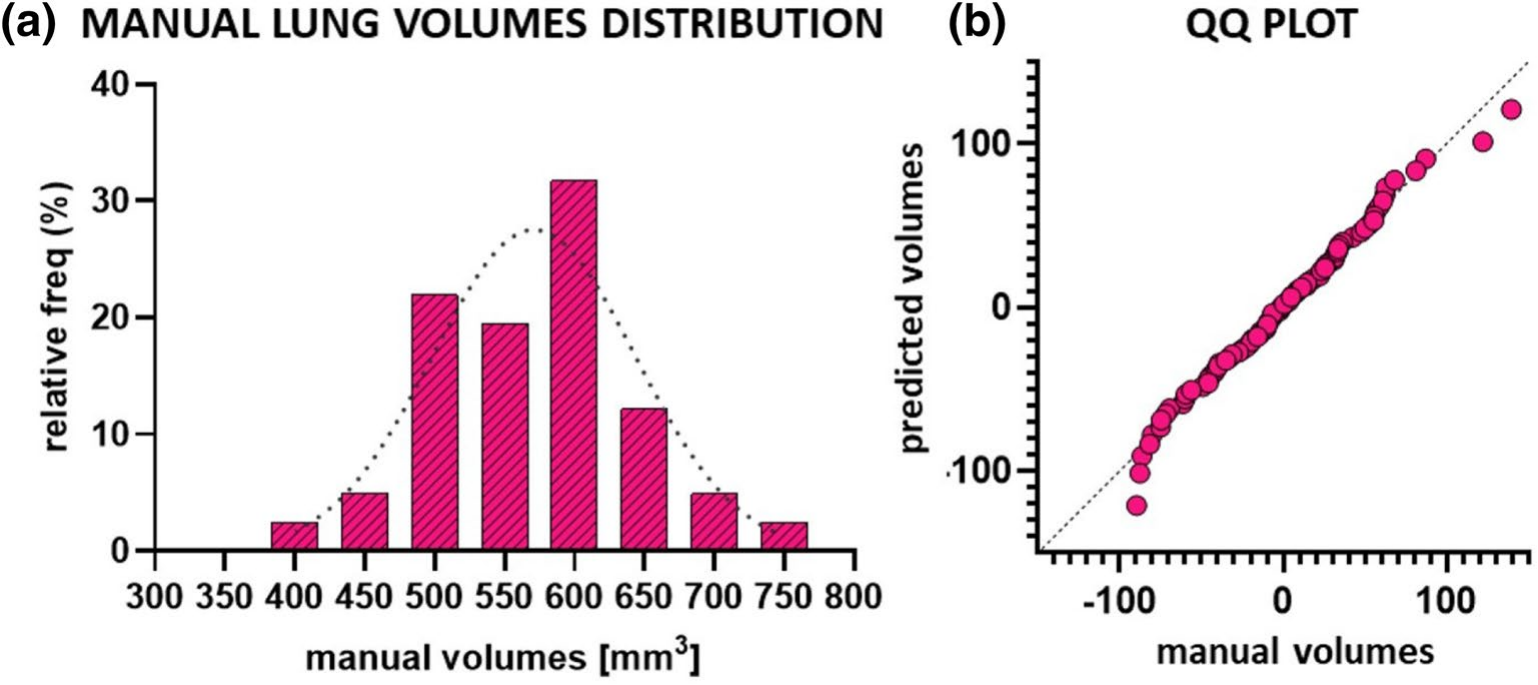
**(a)** Frequency distribution of lung volumes obtained by manual segmentation and used to build up the predicted volume rule (n = 40). A Kolmogorov–Smirnov test with Dallal-Wilkinson-Lillie for P value (p > 0.1) was used as normality test. Mean, standard deviation, 25th and 75th percentiles, lower and upper 95% confidence intervals (CI) were calculated. **(b)** QQ plot between manual and predicted volumes (n = 90). The normality of residuals is confirmed by Anderson–Darling (p = 0.88), D’Agostino-Pearson (p = 0.63), Shapiro–Wilk (p = 0.41) and Kolmogorov–Smirnov (p > 0.1) tests. Data were plotted and analyzed using GraphPad Prism 8 (GraphPad Software, La Jolla, CA, USA), https://www.graphpad.com.

Figure 2a shows the manual lung volume (V_manual_) frequency distribution. As confirmed by a Kolmogorov–Smirnov test with Dallal-Wilkinson-Lillie for P value (p > 0.1), the values are normally distributed. The main parameters of the distribution such as mean (µ), standard deviation (σ), first and third quartiles (25th and 75th percentiles) and 95% confidence interval (CI) were calculated. These parameters were combined as reported in Eq. (1)**,** testing three different pairs of values: A = µ − σ and B = µ + σ, A = 25th percentile and B = 75th percentile, A = − 95% CI and B =  + 95% CI.

1 $$\left\{\begin{array}{c} {\mathrm{V}}_{\mathrm{auto }}< A \to {\mathrm{V}}_{\mathrm{pred }}= A\\ \\ A{<\mathrm{ V}}_{\mathrm{auto }}< B \to {\mathrm{V}}_{\mathrm{pred }}= B\\ \\ {\mathrm{ V}}_{\mathrm{auto }}>B \to {\mathrm{V}}_{\mathrm{pred }}= {V}_{auto}\end{array}\right.$$

Our aim was to obtain a rule to predict the total lung volume for a screened animal (V_pred_), using the volume detected by the automatic segmentation (V_auto_), and avoiding the manual approach. The predicted total lung volume V_pred_ should include, by definition, the severe undetectable lesions. We found that using the 25th percentile and 75th percentile in place of *A* and *B* respectively, V_pred_ provided a robust estimate of the real lung volume. Therefore, we used these two parameters (namely A = 534 mm^3^ and B = 646 mm^3^) to derive V_pred_. Accordingly, the undetectable fibrotic volume, supposed to be lost using automatic segmentation, can be defined as:

2 $${V}_{undetectable}= {V}_{pred }- {V}_{auto }[\mathrm{mm}^3]$$

The QQ plot (Quantile–Quantile plot) in Fig. 2b shows that the residuals, calculated from manual and predicted volumes for about ninety animals (BLM and drug-treated groups), are normally distributed, as confirmed by Anderson–Darling (p = 0.88), D’Agostino-Pearson (p = 0.63), Shapiro–Wilk (p = 0.41) and Kolmogorov–Smirnov (p > 0.1) tests. The differences between manual and predicted methods were calculated to be within 10% of the real volume (V_manual_) for about 80% of animals. As the differences did not exceed 20%, this result was considered acceptable for the purpose of this procedure.

The *‘predictive lung volume method’* has been applied in drug screening studies to give a fast quantification of those severe lesions that would otherwise be lost using semi-automatic segmentation. Further investigations, as reported in the Supplementary Information 1, led to the characterization of the aeration degrees within V_undetectable_(Fig. S1(a))**.** In addition, to confirm the suitability of our model, a retrospective analysis was performed comparing the results obtained using this mathematical rule to the corresponding histological outcomes. As shown in Fig. S1(b) for five independent BLM groups, no significant differences were found between the selected variables (Wilcoxon test, p > 0.05).

### Statistical analysis

The GraphPad Prism 8 software was used for statistical analyses (GraphPad Software, La Jolla, CA, USA)*.* Spearman correlation analysis was used to evaluate the relationship between the selected densitometric indices, histological parameters and micro-CT outcomes. For the comparison between groups in each drug efficacy study, one-way ANOVA with the Dunnett’s post-hoc test for multiple comparisons was used. A 1-tailed Steiger’s Z-test was used to compare dependent correlations. The alpha level of all tests was set at 0.05.

## Results

### Lung densitometry to derive quantitative indices

**Figure 3:**
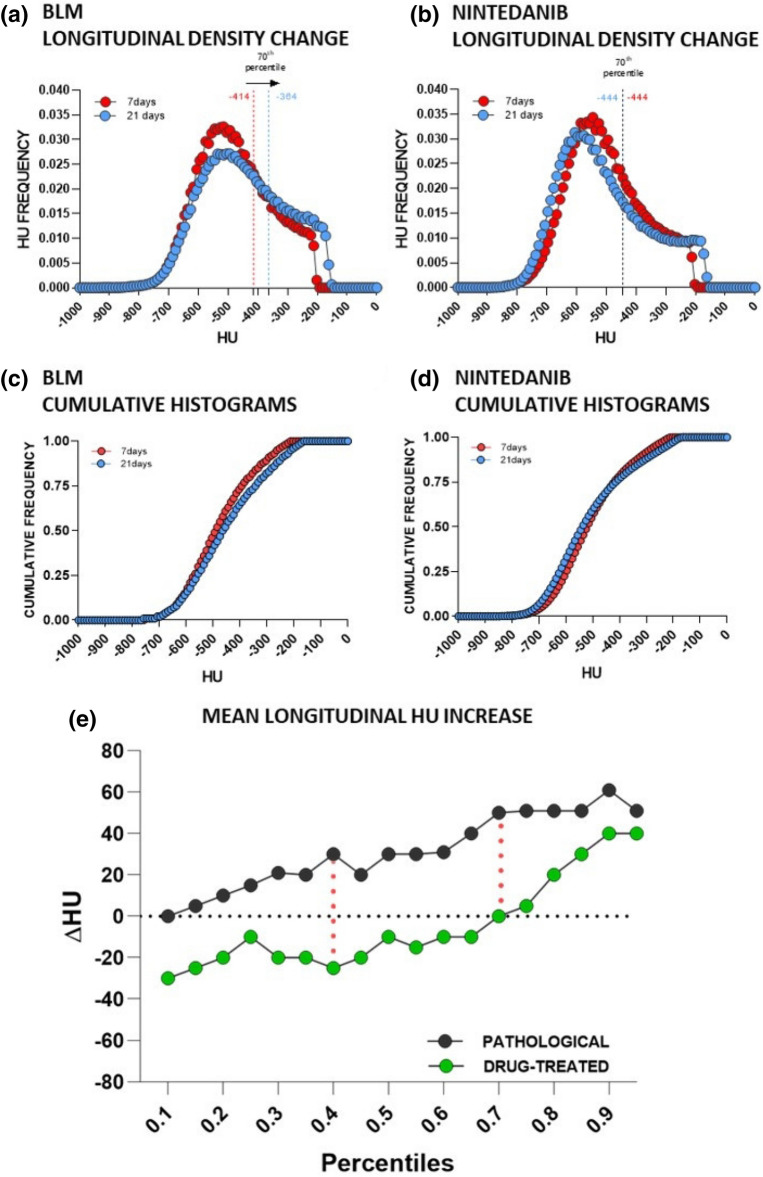
**(a, b)** Mean lung density distributions for BLM and Nintedanib groups at 7 days (red curve) and 21 days (blue curve). The longitudinal variations of the 70th percentile are highlighted (i.e. 50HU for BLM group and no changes for Nintedanib group). **(c, d)** Cumulative mean density histograms for BLM **(b)** and Nintedanib **(c)** groups at 7 days (red lines) and 21 days (blue lines). **(e)** Mean longitudinal HU changes (∆HU) of lung density histograms in the range 10th–90th percentile. As highlighted by dotted red lines, 40th and 70th percentiles show the largest differences between BLM and Nintedanib-treated groups. Figures were created using GraphPad Prism 8 (GraphPad Software, La Jolla, CA, USA), https://www.graphpad.com.

Lung density histograms were acquired for about 100 animals from saline, BLM and Nintedanib-treated groups. The mean distributions for BLM and Nintedanib groups at day 7 and day 21 are reported in Fig. 3a, b, showing the HU longitudinal changes. The corresponding cumulative histograms were subsequently derived at the selected time points (see Fig. 3c, d)**.**

Figure 3e describes the mean HU increase (∆HU) from 7 to 21 days, in each 5th percentile included in the 10th–90th percentile range of lung density histograms for both BLM and Nintedanib-treated groups. The largest HU density differences between groups were detected in the 40th and in the 70th percentiles (∆HU = 55, ∆HU = 50, respectively), as highlighted by dotted red lines. As may be expected, no changes in HU density were observed for saline (data not shown).

**Figure 4:**
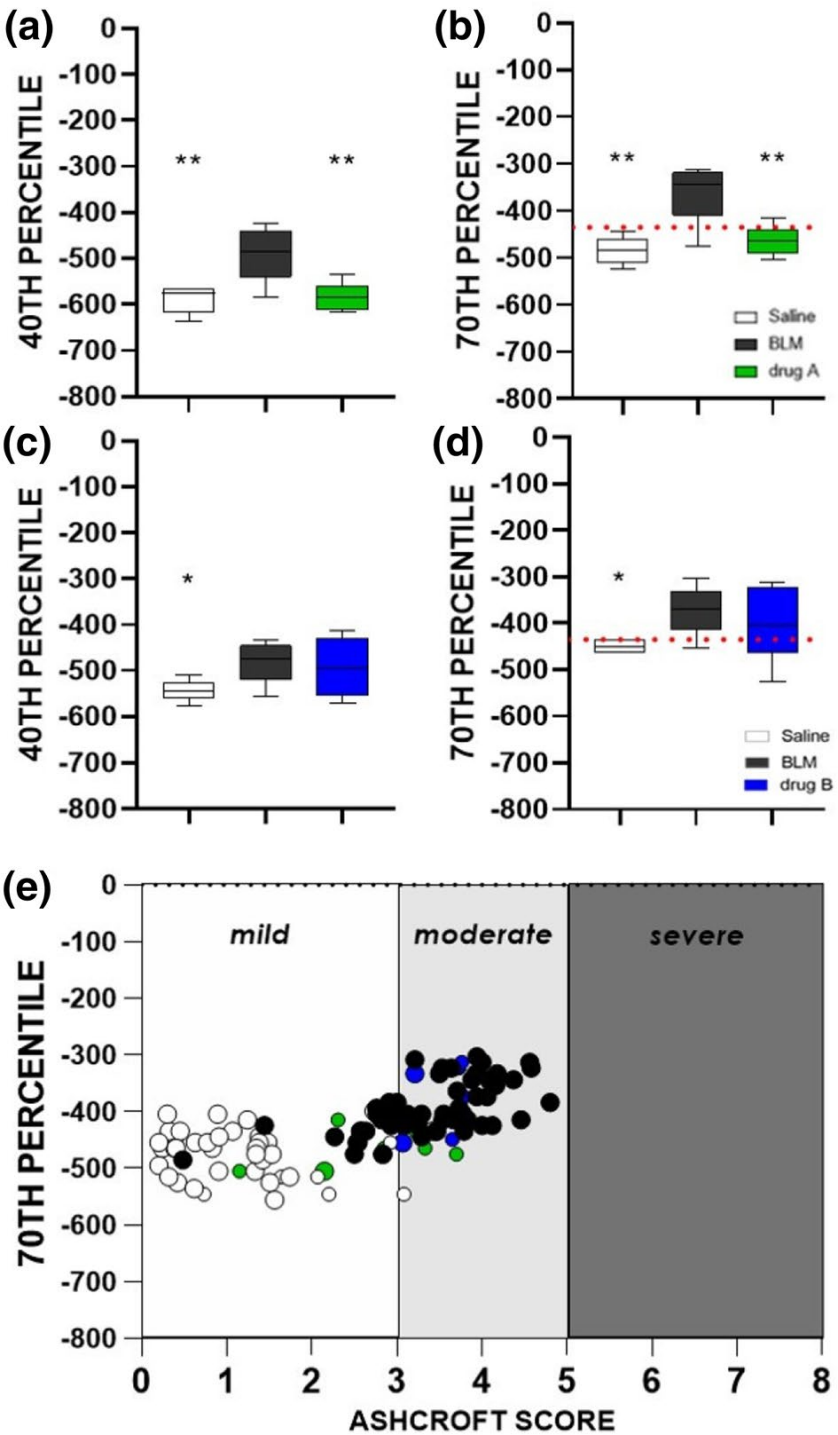
Box and Whiskers plots refer to two drug efficacy studies at 21 days testing drug A, the Nintedanib reference compound (green) **(a, b)** and drug B, a low efficacy candidate (blue) **(c, d).** The central line is the median and the box is defined by 25th and 75th percentiles. Changes in 40th and 70th percentiles for saline and drugs were compared to BLM groups using a one-way ANOVA followed by Dunnett’s test (*p < 0.05; **p < 0.01). The dotted red lines at – 435 HU in the 70th percentile plots represent the threshold value selected as upper limit for normo-aerated region. **(e)** Correlation between selected 70th percentile and Ashcroft score (r_Spearman_ = 0.76, p-value < 0.0001, ****) for vehicles (white) and BLM (black) at 21 days (about n = 100). Green points refer to Nintedanib and blue points refer to the low efficacy treatment used in **(b)** and **(d)**, respectively. Data were plotted and analyzed using GraphPad Prism 8 (GraphPad Software, La Jolla, CA, USA), https://www.graphpad.com.

Figure 4a, b shows the mean values for the 40th and 70th percentiles from an experiment where Nintedanib (i.e. drug A) was used as the reference drug. For both percentiles, statistically significant differences were found between saline and drug A groups compared to BLM (ANOVA followed by Dunnett’s test. **p < 0.01). Figure 4c, d refers to an experiment where the antifibrotic treatment (drug B) did not show any efficacy after examination of classical histological readouts. As expected, significant differences between saline and BLM groups were found for both percentiles, while no differences were found for drug B group when compared to BLM (ANOVA followed by Dunnett’s test. * p < 0.05).

Additionally, the relationship between densitometric and histopathological parameters (i.e. Ashcroft score) was evaluated (about n = 100). The Ashcroft score demonstrated a greater significant correlation with the 70th percentile (*r*_*Spearman*_ = 0.76, *p* < *0.0001*), as compared to the 40th percentile (*r*_*Spearman*_ = 0.69, *p* < *0.0001*), being these correlations with Ashcroft scores significantly different (Z = 4.219, p < 0.0001). This result further confirms the choice to focus on the 70th percentile, plotted against Ashcroft score in Fig. 4e. We considered three levels of fibrotic disease based on histopathological data: normal lungs with mild parenchymal changes were associated with an Ashcroft score of 0–3, while moderate and severe pathologic abnormalities reflected scores of 4 and 5–8, respectively^37^. As highlighted, 94% of healthy animals (white) (n = 47) are included in the mild range and 77% of the pathological ones (black) in the moderate region (n = 57), whilst no severe scores (Ashcroft ≥ 5) were observed. This evidence suggests a strong alignment of the selected index with the histological findings, as further confirmed by the distribution of drug A (green) and drug B (blue) in the plot, with the reference drug almost completely confined to the mild region and the low efficacy compound totally confined to the moderate.

### Definition of aerated lung regions: the new preclinical thresholds

As clearly shown in Fig. 4b, d and confirmed by the linear regression with Ashcroft score in Fig. 4e, the 70th percentile can discriminate between animals characterized by heterogeneous fibrotic pulmonary lesions. As a step forward, we used the value – 435 HU (red dotted lines in Fig. 4b, d), namely the mean between the lowest 70th percentile in BLM groups and the highest 70th percentile in saline groups (n = 100), to define lung aeration regions in mice.

**Figure 5:**
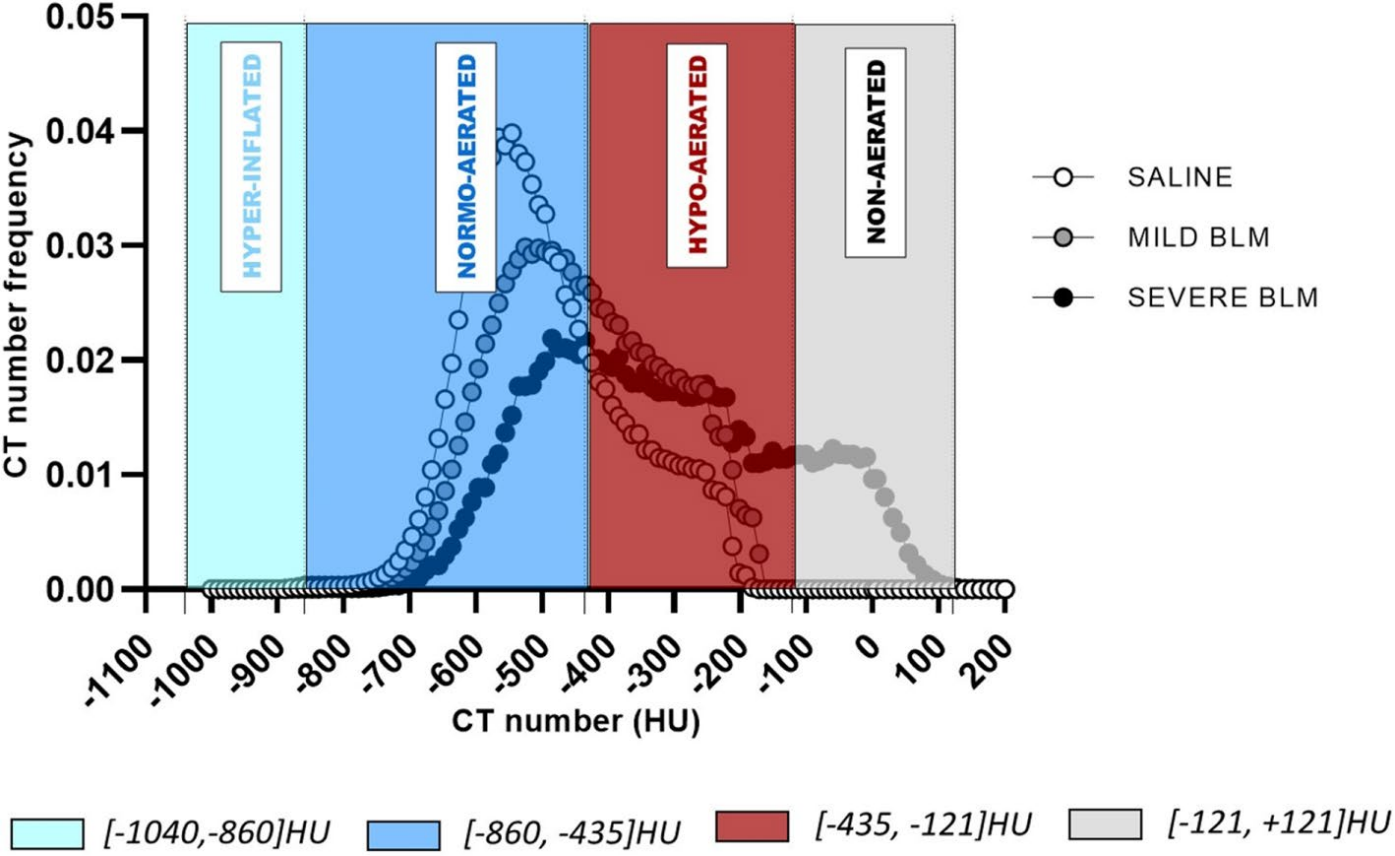
CT number frequency distributions describing a normal condition (saline-white points) and two pathological-fibrotic conditions, mild BLM (grey points) and severe BLM (black points). The lung aeration compartments defined by the new HU preclinical ranges are overlapped: in light blue the hyper-inflated [− 1,040, − 860] HU, in blue the normo-aerated [− 860, − 435] HU, in red the hypo-aerated [− 435, − 121] HU and in grey the non-aerated [− 121, + 121] HU. Figure was created using GraphPad Prism 8 (GraphPad Software, La Jolla, CA, USA), https://www.graphpad.com.

The representative HU frequency distributions for saline and BLM with mild and severe histological lesions, are reported in Fig. 5. 70% of the AUC (area under curve) for the normal murine lung (white curve) is included in the range [− 1,000, − 435] HU. This supports the decision to fix the upper limit for the normo-aerated compartment at – 435 HU. The lower limit was set at – 860 HU, since only 0.02% of the AUC (mean value over 100 subjects) was included in the interval [− 1,040, − 860] HU. Following the same procedure, the hypo-aerated region was defined by fixing its upper limit at – 121 HU, namely the highest HU value reached in lung density histograms using automatic segmentation (n = 100). Finally, to determine the external hyper-inflated (not considered in this work) and non-aerated compartments, we referred to Gattinoni et al.^38^ . We centered these regions at -950HU and at 0HU respectively, in order to reflect their theoretical air/tissue compositions: 95% air + 5% tissue for the first and 100% tissue for the latter. Consequently, we limited the hyper-inflated compartment to the range [− 1,040, − 860] HU and the non-aerated compartment to the range [− 121, + 121] HU.

The grey curve in Fig. 5 represents the HU frequency distribution for a BLM animal with mild/moderate fibrotic lesions and it is totally included in the range [− 860, − 121] HU. This justifies the use of the semi-automatic segmentation for the OA experiments described herein. Indeed, when more severe fibrotic lesions are revealed (black curve), as experienced with 2–3% of the animals, the distribution spreads across three different compartments, ranging from – 860 HU to + 121 HU. In these cases, in order to detect the volume included in the non-aerated compartment, a manual segmentation is needed. As described in “Methods”section, the predictive lung volume method represents an alternative rule to estimate, if any, the fibrotic volume in the region [− 121, + 121] HU. This method, applied to all the experimental groups we need to compare, can give a reliable quantification of the undetectable fibrotic volumes (see Supplementary Materials 1 for a detailed description of tissue aeration degrees within V_undetectable_).

As detailed above, the new preclinical ranges allow discrimination between different lung regions based on their air content. In order to test if these new thresholds could better describe the heterogeneity of lung fibrosis in mice compared to clinical ones^7^, a retrospective analysis was performed on 250 mice from several independent experiments. A double thresholding procedure was carried out and micro-CT parameters were extracted and compared for all the screened animals.

**Figure 6:**
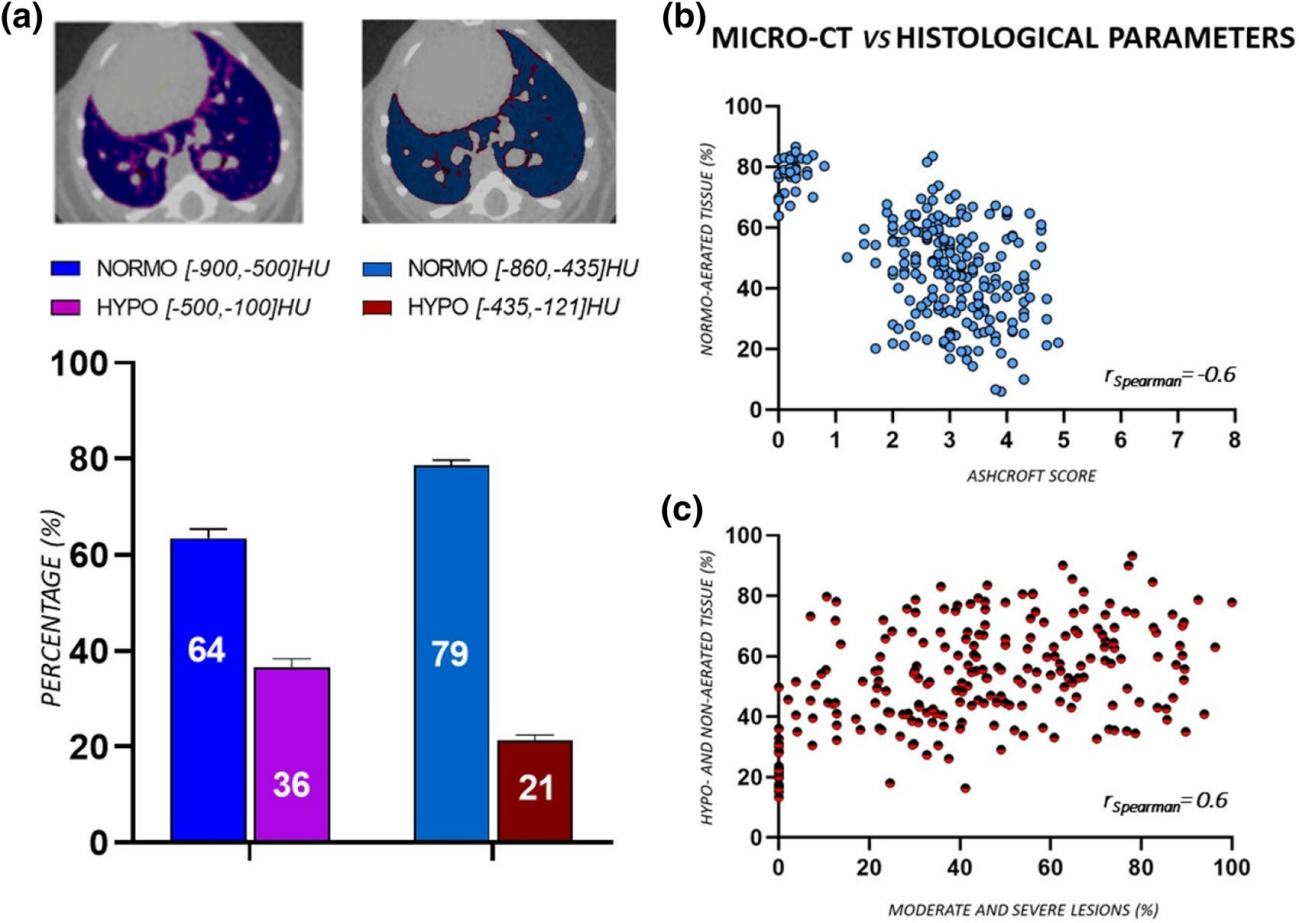
**(a)** The mean percentages of normo- and hypo-aerated tissues for healthy mice (n = 23), using both clinical and preclinical lung aeration thresholds. The improvement in normo-aerated compartment with the new thresholds, from 64 to 79%, reflects physiological conditions (each variable is represented as mean ± s.e.m). Axial micro-CT images of the same healthy mouse show the differences between clinical and preclinical thresholds applied to the segmented lung. **(b, c)** Relationship between histological and micro-CT parameters, obtained using preclinical thresholds (n = 250). A good correlation was found comparing % normo-aerated tissue against Ashcroft score (r_Spearman_ = − 0.6 (p-value < 0.0001, ****)) and %hypo- and non-aerated tissues against the %moderate and severe lesions (r_Spearman_ = 0.6 (p-value < 0.0001, ****)). Analyze 12.0 (Mayo Clinic, Rochester, MN) was used to process μCT datasets, https://www.analyzedirect.com. Data were plotted and analyzed using GraphPad Prism 8 (GraphPad Software, La Jolla, CA, USA), https://www.graphpad.com.

The mean percentages of normo- and hypo-aerated tissue for healthy mice, obtained using both clinical and preclinical ranges, are reported in Fig. 6a. The introduction of new HU thresholds implies a mean increase of 15% in normo-aerated volume with respect to clinical ranges. Accordingly, applying preclinical density thresholds, physiological conditions are mimicked.

The antifibrotic effect evaluation in preclinical studies relies on invasive ex-vivo measurements, involving labor-intensive biochemical analysis and/or histological scoring. Although longitudinal studies are obviously precluded, this analysis is considered a gold standard procedure. On the other hand, i*n-vivo* microtomographic readouts refer to the restoration of the normo-aerated region (i.e. increased normo-aerated volume) or to the reduction of the less aerated compartments (i.e. hypo-aerated and non-aerated volumes). Therefore, the volume of each compartment constituting the whole lung volume (i.e. hyper-, normo-, hypo- and non-aerated) impacts on drug efficacy evaluation by micro-CT. To verify the reliability and suitability of the preclinical density thresholds, ex-vivo and in-vivo efficacy results were compared for a total of 250 mice from the above-mentioned drug discovery preclinical trials including saline, BLM and drug-treated groups (i.e. 13 antifibrotic candidates).

Normo-aerated, hypo-aerated and non-aerated tissues were evaluated by applying either preclinical or clinical ranges and the corresponding volumes correlated to total Ashcroft scores and moderate and severe fibrotic lesions (Fig. 6b, c). The correlation coefficients, compared to clinical ranges, showed an increase from r_Spearman_ = − 0.5 (p-value < 0.0001, ****) to r_Spearman_ = − 0.6 (p-value < 0.0001, ****) (Fig. 6b) and from r_Spearman_ = 0.5 (p-value < 0.0001, ****) to r_Spearman_ = 0.6 (p-value < 0.0001, ****) (Fig. 6c)**.**

As shown in Fig. S2a, b, higher correlation coefficients were observed focusing on healthy and pathological groups only and excluding the antifibrotic candidates from the analysis. The improvement using preclinical thresholds was confirmed (see Supplementary Information 1 for more details).

## Discussion

Pulmonary CT imaging is widely used for diagnostic purposes, playing a crucial role in clinical practice^3^. A huge community of physicians and radiologists is increasingly committed to the creation and deployment of novel tools, protocols and algorithms^39,40^. In contrast, the critical mass is still missing and use is very fragmented in the preclinical setting. Micro-CT users, mostly from academic centers, usually deal with only a limited number of mice. In fibrosis drug discovery, however, micro-CT imaging needs to be applied to a large number of mice and deliver reliable data in a very short time to support the commitment to find the best anti-fibrotic drug candidates for clinical development. To date, a robust validation of micro-CT in pulmonary fibrosis drug discovery has not been performed, hence its application in this field has never been described. As detailed above, the validation process required the employment of fibrotic mice, the pathology being induced with a well standardized protocol and pharmacological treatment carried out including both standard reference drugs (i.e. Nintedanib) and non-responsive compounds. Micro-CT imaging was always performed using established acquisition parameters and the samples for the histological examination were carefully examined by fully trained histopathologists. In this work, we evaluated the use of micro-CT density measurements in a murine model of BLM-induced lung fibrosis, defining reference values for the quantification of longitudinal changes in lung parenchyma.

**Figure 7:**
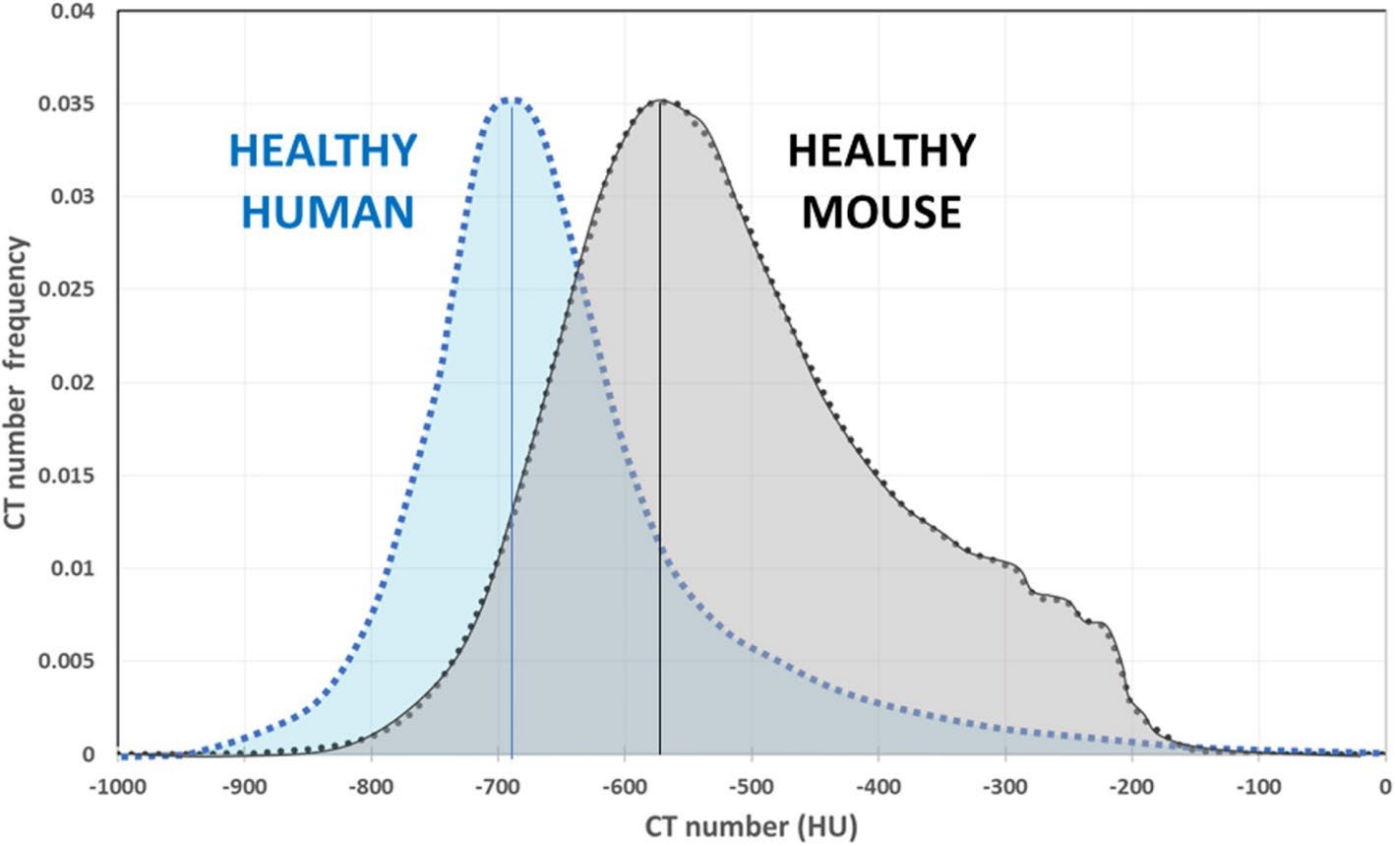
HU lung density distributions in normal conditions, for human at functional residual capacity (blue area) and anesthetized mouse at the end of expiration phase (grey area).

Contrary to the clinical setting^4^, the impossibility of revealing morphological features linked to fibrotic disease (such as ground glass and reticular opacities, honey-combing, consolidations)^13,41^, prevents the identification of any correspondence between high- or low-density parenchymal areas and texture abnormalities, thus excluding visual score determination. In addition, to the best of our knowledge, although micro-CT metrics such as aerated lung volume and tissue lung volume are recognized as primary outcomes in murine models of lung diseases^21^, no attempts have been made so far to fix HU limits for lung aeration compartments. In clinical setting, most authors refer to four lung regions with different aeration distinguishing between hyper-inflated, normally-aerated, hypo-aerated and non-aerated compartments^7^. Despite the use of these thresholds for microtomographic images quantification^24^, the translation of clinical cut-off values to preclinical applications, could lead to misinterpretation or improper results. The need for a preclinical characterization is evident when comparing human and mouse HU density histograms. As shown in Fig. 7, in normal patients at functional residual capacity (blue area), about 50% of the total lung volume is included in the compartment [− 800, − 600] HU^7^. The grey area represents instead the average distribution over about 50 healthy mice at the end-expiration phase. The curve peaks at approximately -600HU, with a shift of about 100 units and a broader frequency component towards higher HU values compared to human healthy lung. Human CT acquisitions in fact, can be performed at total lung capacity (TLC) or at functional residual capacity (FRC)^9,40^ whereas, to address free breathing variabilities, mice imaged by micro-CT are always sedated and subjected to respiratory gating procedures. Anesthesia is a crucial factor influencing the quality of micro-CT scans. Although the anesthetic procedures are well standardized with a fixed time of induction and a constantly monitored breathing rate (100–120 breaths per minute)^42^, respiratory motion represents a challenge especially at the boundary between lung and diaphragm. Even in healthy mice, about 20% of total lung voxels show HU > -435 and are likely affected by either movement artifacts or noise, in contrast to human lung density distribution (Fig. 7). Taken together, these evidences prompt us to derive the best densitometric indices reflecting the fibrosis disease evolution in mice, deriving lung aeration levels suitable for developing preclinical density thresholds.

Preclinical density thresholds distinguish between four lung compartments with different air content, as reported by Gattinoni et al. for clinical CT^7^. The new HU ranges allow the properly reproduction of physiological conditions, given that the percentage of hypo-aerated tissue in saline is reduced compared to clinical ranges (Fig. 6a). This result is confirmed whatever the severity of the employed model. In fact, the cut-off value used as starting point to derive the preclinical thresholds (i.e. – 435 HU) (Fig. 4b–d), ensures their suitability for more severe fibrotic models. In addition, even if the hyperinflated compartment was not revealed in the BLM model described herein, this region could be useful for the detection of emphysema in mice^12^.

The retrospective analysis, as expected, confirmed that preclinical thresholds are reliable for performing drug efficacy evaluations. To support this result, the comparison between histological scores and micro-CT parameters obtained by applying either preclinical or clinical ranges, underlined an improvement in correlation coefficients using new thresholds. The good correlation obtained, albeit comparing a 3D measurement with a 2D snapshot of the pathology (Fig. 6b, c), corroborated the pivotal role of micro-CT imaging in drug discovery. In fact, despite histology still represents the gold standard for evaluating lung fibrosis progression, the histological outcomes refer to less than 1% of the total lung volume. The comprehensive evaluation given by micro-CT analysis could then provide a more reliable estimate of antifibrotic drug efficacies, reducing the sample size planned in power analysis tests (see Supplementary Information 1). Furthermore, histology does not allow longitudinal studies and its time-consuming nature and vulnerability to operator-dependent errors make the technique unsuitable for handling a large number of animals. The agreement with histological findings, also confirmed that density changes in selected percentiles (40th and 70th percentiles) can reflect a pathological lung condition (Fig. 4e) and its evolution over time. These measurable readouts were obtained from HU density distributions, reflecting the longitudinal changes between 7 and 21 days. It is also noteworthy that the use of Nintedanib as a reference compound in the time-course study ensured the attainment of maxima differences compared to the pathological group.

Two basic protocols were described to extract lung segmentation maps. Although the manual segmentation was used only for a negligible percentage of animals with severe fibrotic lesions, we developed a fast alternative mathematical method to predict lung volumes in drug screening. This procedure provides a rapid quantification of denser volumes, otherwise undetectable through the automatic approach. Despite not being an objective of this work, the *‘predictive lung volume method’* shows a great potential, especially considering that a drug discovery program may require about 800–1,000 animals/year to be screened, a number unmanageable using a manual procedure. Nevertheless, the integration of advanced algorithms of deep learning, computer vision or active shape models in image post-processing could open the way to a completely automatic densitometric investigation, where assessment of fibrosis and compounds evaluation will be fully guided by micro-CT.

## Supplementary information

Supplementary Information.

Supplementary file2

Supplementary file3

Supplementary file4

Supplementary Video 1.

Supplementary Video 2.

## References

[1] Raghu G (2011). An official ATS/ERS/JRS/ALAT statement: Idiopathic pulmonary fibrosis: Evidence-based guidelines for diagnosis and management. Am. J. Respir. Crit. Care Med..

[2] Silva, M., Milanese, G., Seletti, V., Ariani, A. & Sverzellati, N. Pulmonary quantitative CT imaging in focal and diffuse disease: Current research and clinical applications. *Br. J. Radiol.* **91** (2018). 10.1259/bjr.20170644 PMC5965469 29172671

[3] Ball L, Vercesi V, Costantino F, Chandrapatham K, Pelosi P (2017). Lung imaging: How to get better look inside the lung. Ann. Transl. Med..

[4] Sverzellati N (2007). Score visivo e indici di TC quantitativa nella fibrosi polmonare: Correlazioni con i dati di compromissione funzionale. Radiol. Med..

[5] Mascalchi M, Camiciottoli G, Diciotti S (2017). Lung densitometry: Why, how and when. J. Thorac. Dis..

[6] Gattinoni L, M. D. *et al.* Adult respiratory distress syndrome profiles by computed tom. *J. Thorac. Imaging* (1986). 10.1097/00005382-198607000-00005 3298678

[7] Gattinoni L, Caironi P, Pelosi P, Goodman LR (2001). What has computed tomography taught us about the acute respiratory distress syndrome?. Am. J. Respir. Crit. Care Med..

[8] Gattinoni L (1991). CT scan in ARDS: Clinical and physiopathological insights. Acta Anaesthesiol. Scand..

[9] Vieira SRR (1998). A lung computed tomographic assessment of positive end-expiratory pressure-induced lung overdistension. Am. J. Respir. Crit. Care Med..

[10] Stoel BC, Stolk J (2004). Optimization and standardization of lung densitometry in the assessment of pulmonary emphysema. Invest. Radiol..

[11] Nakagawa H (2019). Quantitative CT analysis of honeycombing area predicts mortality in idiopathic pulmonary fibrosis with definite usual interstitial pneumonia pattern: A retrospective cohort study. PLoS ONE.

[12] Parr DG (2009). Exploring the optimum approach to the use of CT densitometry in a randomised placebo-controlled study of augmentation therapy in alpha 1-antitrypsin deficiency. Respir. Res..

[13] Moore BB (2013). Animal models of fibrotic lung disease. Am. J. Respir. Cell Mol. Biol..

[14] Clark DP, Badea CT (2014). Micro-CT of rodents: State-of-the-art and future perspectives. Phys. Med..

[15] Gammon, S. T. *et al.* Preclinical anatomical, molecular, and functional imaging of the lung with multiple modalities. *Am. J. Physiol. Lung Cell. Mol. Physiol.* **306** (2014). 10.1152/ajplung.00007.2014 24658139

[16] Bidola P (2019). A step towards valid detection and quantification of lung cancer volume in experimental mice with contrast agent-based X-ray microtomography. Sci. Rep..

[17] Saito S, Murase K (2012). Detection and early phase assessment of radiation-induced lung injury in mice using micro-CT. PLoS ONE.

[18] Ninaber MK (2015). Lung structure and function relation in systemic sclerosis: Application of lung densitometry. Eur. J. Radiol..

[19] Perez JR (2017). A comparative analysis of longitudinal computed tomography and histopathology for evaluating the potential of mesenchymal stem cells in mitigating radiation-induced pulmonary fibrosis. Sci. Rep..

[20] Colombi D (2015). Research article: Visual vs fully automatic histogram-based assessment of idiopathic pulmonary fibrosis (IPF) progression using sequential multidetector computed tomography (MDCT). PLoS ONE.

[21] Bell RD, Rudmann C, Wood RW, Schwarz EM, Rahimi H (2018). Longitudinal micro-CT as an outcome measure of interstitial lung disease in TNF-transgenic mice. PLoS ONE.

[22] Mah K, Van Dyk J (1988). Quantitative measurement of changes in human lung density following irradiation. Radiother. Oncol..

[23] Johnson KA (2007). Imaging techniques for small animal models of pulmonary disease: Micro-CT. Toxicol. Pathol..

[24] Reske AW (2011). Extrapolation in the analysis of lung aeration by computed tomography: A validation study. Crit. Care.

[25] de Langhe, E. *et al.* Quantification of lung fibrosis and emphysema in mice using automated micro-computed tomography. *PLoS One* **7** (2012). 10.1371/journal.pone.0043123 PMC3418271 22912805

[26] Ask K (2008). Comparison between conventional and ‘clinical’ assessment of experimental lung fibrosis. J. Transl. Med..

[27] Degryse AL, Lawson WE (2011). Progress toward improving animal models for Ipf. Am. J. Med. Sci..

[28] Ruscitti, F., Ravanetti, F., Donofrio, G., Ridwan, Y., van Heijningen, P., Essers, J., Villetti, G., Cacchioli, A., Vos, W., Stellari, F. F. A multimodal imaging approach based on micro-CT and fluorescence molecular tomography for longitudinal assessment of bleomycin-induced lung fibrosis in mice. *J. Vis. Exp* **134** (2018). 10.3791/56443 PMC5933503 29708527

[29] Wollin L, Maillet I, Quesniaux V, Holweg A, Ryffel B (2014). Antifibrotic and anti-inflammatory activity of the tyrosine kinase inhibitor nintedanib in experimental models of lung fibrosiss. J. Pharmacol. Exp. Ther..

[30] Bayne, K. Revised guide for the care and use of laboratory animals available. American Physiological Society*. Physiologist* **39** (1996). 8854724

[31] De Vooght, V., Vanoirbeek, J.A.J., Haenen, S., Verbeken, E., Nemery, B. H. P. Oropharyngeal aspiration: an alternative route for challenging in a mouse model of chemical-induced asthma. *Toxicology* 84–89 (2009). 10.1016/j.tox.2009.02.007 19428947

[32] Barbayianni I, Ninou I, Tzouvelekis A, Aidinis V (2018). Bleomycin revisited: A direct comparison of the intratracheal micro-spraying and the oropharyngeal aspiration routes of bleomycin administration in mice. Front. Med..

[33] Ashcroft T, Simpson JM, Timbrell V (1988). Simple method of estimating severity of pulmonary fibrosis on a numerical scale. J. Clin. Pathol..

[34] Hübner RH (2008). Standardized quantification of pulmonary fibrosis in histological samples. Biotechniques.

[35] Ruscitti F (2017). Longitudinal assessment of bleomycin-induced lung fibrosis by micro-CT correlates with histological evaluation in mice. Multidiscip. Respir. Med..

[36] Meganck JA, Liu B (2017). Dosimetry in micro-computed tomography: A review of the measurement methods, impacts, and characterization of the quantum GX imaging system. Mol. Imaging Biol..

[37] Stellari, F. F. *et al.* Heterologous matrix metalloproteinase gene promoter activity allows in vivo real-time imaging of bleomycin-induced lung fibrosis in transiently transgenized mice. *Front. Immunol.* **8** (2017). 10.3389/fimmu.2017.00199 PMC5331072 28298912

[38] Gattinoni L (1988). Relationships between ung computed tomographic density, gas exchange, and PEEP in acute respiratory failure. Anesthesiology.

[39] Walsh SLF, Calandriello L, Silva M, Sverzellati N (2018). Deep learning for classifying fibrotic lung disease on high-resolution computed tomography: A case-cohort study. Lancet Respir. Med..

[40] Bodduluri S, Reinhardt JM, Hoffman EA, Newell JD, Bhatt SP (2018). Recent advances in computed tomography imaging in chronic obstructive pulmonary disease. Ann. Am. Thorac. Soc..

[41] Uhl, F. E., Wagner, D. E. & Weiss, D. J. Preparation of decellularized lung matrices for cell culture and protein analysis. *Methods Mol. Biol.* **1627** (2017). 10.1007/978-1-4939-7113-8_18 PMC7456164 28836208

[42] Grimaud J, Murthy VN (2018). How to monitor breathing in laboratory rodents: a review of the current methods. J. Neurophysiol..

