# Supplementary material for: In-vivo lung fibrosis staging in a bleomycin-mouse model: a new micro-CT guided densitometric approach
Source: Sci Rep. 2020 Oct 30;10:18735. doi: 10.1038/s41598-020-71293-3 (PMC7603396; doi:10.1038/s41598-020-71293-3)
Supplement: Supplementary file 1 — Supplementary Information. [file 41598_2020_71293_MOESM1_ESM.docx]

*In-vivo* lung fibrosis staging in a bleomycin-mouse model: a new micro-CT guided densitometric approach

*Laura Mecozzi^1^, Martina Mambrini^2^, Francesca Ruscitti^3^, Erica Ferrini^2^, Roberta Ciccimarra^2^, Francesca Ravanetti^2^, Nicola Sverzellati^1^, Mario Silva^1^, Livia Ruffini^4^, Sasha Belenkov^5^, Maurizio Civelli^3^, Gino Villetti^3^, Fabio Franco Stellari^3^*.*

*^1^ Department of Medicine and Surgery, University of Parma, Parma, Italy*

*^2^ Department of Veterinary Science, University of Parma, Parma, Italy*

*^3^ Chiesi Farmaceutici S.p.A., Corporate Pre-Clinical R&D, Parma, Italy*

*^4^ Department Nuclear Medicine, Academic Hospital of Parma, Parma, Italy*

*^5^ Perkin Elmer, Inc., Waltham, MA, USA*

**SUPPLEMENTARY INFORMATION**

1. **Predictive lung volume method: within the undetectable volume**

The undetectable volume was quantified for 40 animals (previously analyzed by manual segmentation), using the **Equation (2)**. To deepen the undetectable volume characterization, we studied the tissue aeration degrees within **V_undetectable_**. We considered that normo-aerated tissue could never be included in this volume: if present, the normo-aerated tissue should always belong to the automatically detectable parenchyma. Therefore, we evaluated the ratio of hypo-aerated and non-aerated tissues constituting **V_undetectable_**. Referring to the manually segmented volumes, we explored the correlation between non-aerated tissues and the corresponding undetectable volumes for each subject, as derived using **Equation (2).** An excellent correlation was found between the two variables (R^2^=0.98) (**Fig.S1(a)**)**,** suggesting a precise lung tissue composition within the undetectable volume. Specifically, the non-aerated tissue and the hypo-aerated tissue, as calculated using preclinical thresholds, should be present in a ratio equal to 2.33 (n=25) (**Fig.S1(a)**). This ratio was demonstrated to decrease to 1.78 when clinical thresholds^7^ were used to quantify non-aerated and the hypo-aerated tissues (data not shown).

To validate the proposed predictive rule, a comparison with histological outcomes was made. Five independent BLM groups were used to extract hypo- and non-aerated volumes (using the predicted volume method) and moderate and severe degrees (by histopathological characterization). These parameters, as reported in **Fig.S1(b),** were compared and no significant differences were found for each BLM group (Wilcoxon test, p > 0.05).

Further investigations are ongoing to obtain a more precise extraction of the undetectable volume, using *ad hoc* algorithms for the whole lung automatic segmentation. Accordingly, detailed HU frequency distributions should be provided including the non-aerated compartment, thus opening the route to a fully standalone micro-CT densitometric analysis.

1. **Clinical and preclinical thresholds: comparison with histological outcomes**

Focusing on healthy and pathological groups, a retrospective analysis across 85 mice was made comparing e*x-vivo* and *in-vivo* efficacy results. The correlation between the %normo-aerated_preclinical_ tissue and the Ashcroft scores showed an increase from r_Spearman_= -0.7 (p-value <0.0001****) to r_Spearman_= -0.8 (p-value <0.0001****) with respect to clinical thresholds **(Fig.S2(a)).** In addition, the linear correlation coefficient increases from R^2^=0.7 to R^2^=0.8 using preclinical thresholds. The same increase arose comparing the %hypo- and non-aerated_preclinical_ tissue to the %moderate and severe degrees, from R^2^=0.6 up to R^2^=0.7(**Fig.S2(b)**).

As shown in **Fig.6(b-c)**, lower correlation coefficients were observed when all the animals from saline, BLM and drug-treated groups (n=250, including 13 anti-fibrotic candidates), were pulled together. Anyway, the improvements observed using preclinical thresholds were confirmed.

1. **Power analysis to plan experiments: histological vs micro-CT parameters**

The experiments planning relies on an a priori power analysis (*G*Power tool 3.1.9.4*) based on the expected pharmacological effect in terms of histological outcomes (i.e. Ashcroft score variations between pathological-BLM and drug-treated groups). Micro-CT parameters (including any value derived from predicted lung volumes or from densitometric analyses) are not taken into consideration to this purpose. However, despite histology is considered the gold standard technology for evaluating lung fibrosis progression in drug screening routine, the histological outcomes refer only to a negligible region of the lung (< 1%). The 3D micro-CT analysis gives instead a comprehensive evaluation of the whole lung volume conditions and, we can suppose, a more representative quantification of the investigated antifibrotic drugs efficacies. Therefore, we might expect that using micro-CT parameters as indices of the pharmacological effect, the power analysis should estimate a reduced number of mice. To support this assumption, we used two representative experiments (two different antifibrotic candidates) to retrospectively compare the outputs of the power analysis tool (see **Table S1**).

We simulated an experiment’s planning (effect size determination from group parameters) by fixing the power (0.8) and introducing as effect size factors: Ashcroft scores, 70^th^ percentiles and predicted %poorly-aerated tissue (i.e. predicted hypo-aerated and non-aerated tissues).

As expected, using micro-CT outcomes, either predicted or densitometric values, the estimated sample size for both experiments is substantially reduced by almost one-half. This result could be of great interest and impact, giving the idea of the potentialities of the technique.


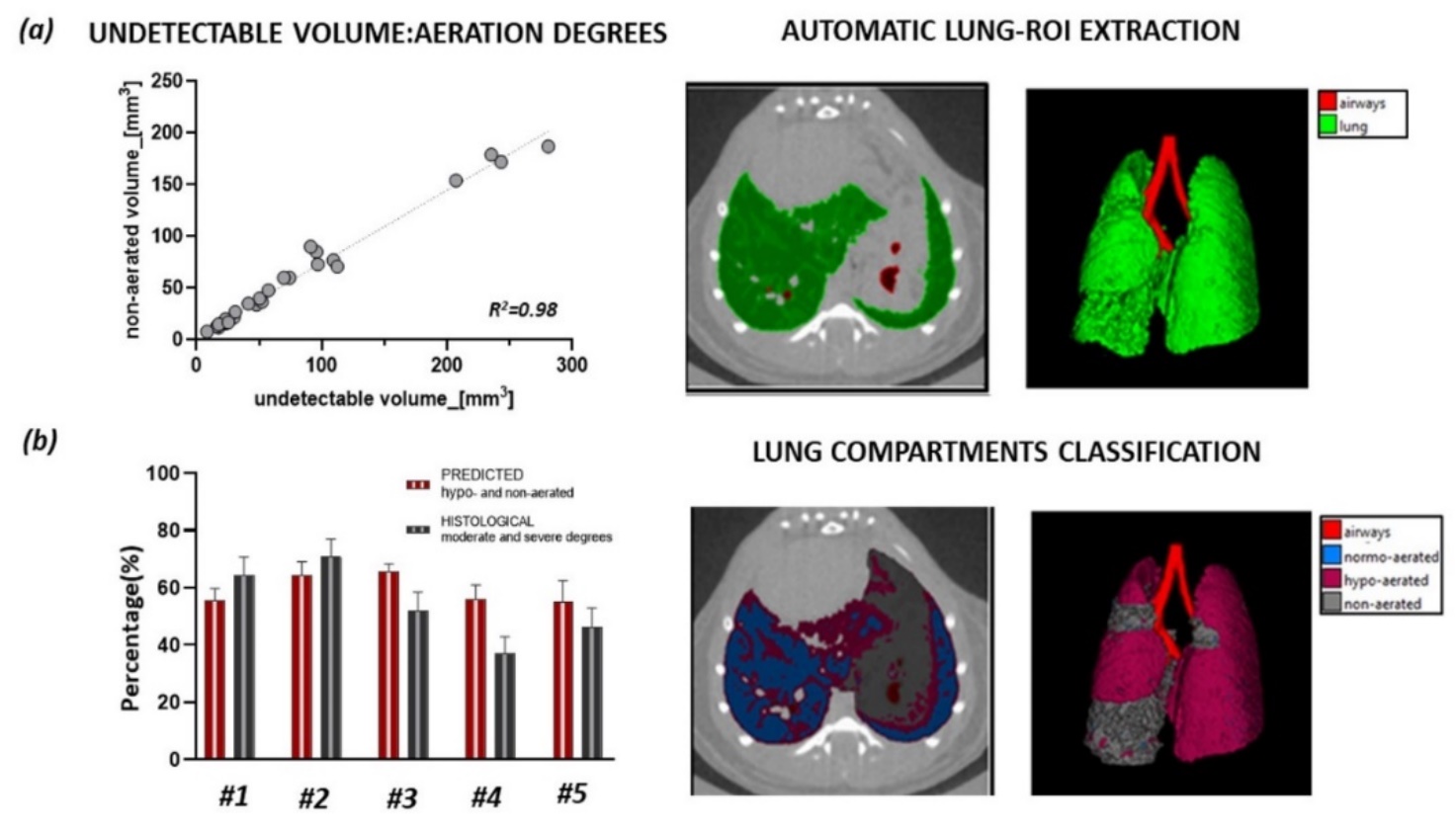
***Figure S1:*** ***(a)*** *Relationship between undetectable volumes and non-aerated tissue volumes for about 30 mice. The results obtained using preclinical thresholds (non-aerated region: [-121, +121]HU), show that 70% of the undetectable volume corresponds to non-aerated tissue. Representative 3D lung reconstructions for a fibrotic mouse: the automatic lung segmentation (green ROI)* ***(top)*** *and the manual segmentation procedure* ***(bottom)****. The compartments with different aeration degrees are classified using preclinical thresholds* ***(b)*** *Predicted hypo- and non-aerated percentages are compared to the corresponding percentages of moderate and severe histological degrees. Five independent BLM groups are shown. For each pair of values, no statistical differences were found (each variable is represented as mean ± s.e.m) (Wilcoxon test, p > 0.05). Analyze 12.0 (Mayo Clinic, Rochester, MN*) *was used for μCT data analyses,*[*www.analyzedirect.com*](http://www.analyzedirect.com) *Data were plotted and analyzed using GraphPad Prism 8 (GraphPad Software, La Jolla, CA, USA),* [*www.graphpad.com*](http://www.graphpad.com/)*.*


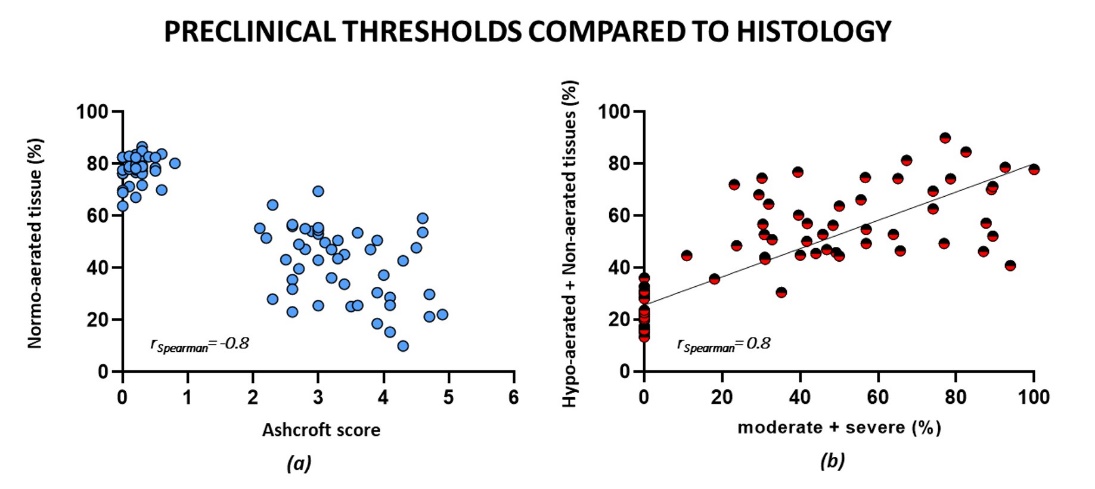
***Figure S2:*** *Comparison between new preclinical thresholds and histological outcomes for saline and BLM groups (n=85).* ***(a)*** *The relationship between the % normo-aerated tissue and Ashcroft scores reveals an increase in the linear correlation coefficient from R^2^=0.7 using clinical ranges to R^2^=0.8 with preclinical thresholds* ***(b)*** *The %moderate and severe histological degrees is compared to the %hypo- and non-aerated tissues showing a highly significant correlation (r_Spearman_= 0.83, p-value <0.0001, ****). Data were plotted and analyzed using GraphPad Prism 8 (GraphPad Software, La Jolla, CA, USA),* [*www.graphpad.com*](http://www.graphpad.com/)*.*

***
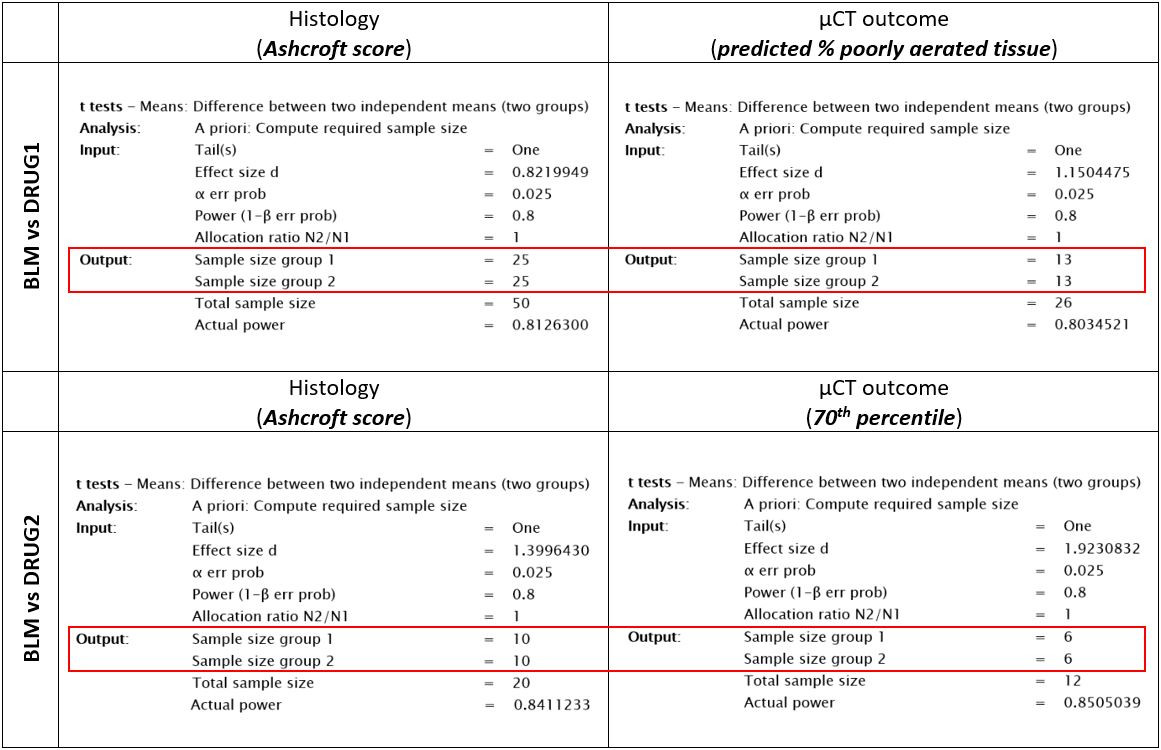
Table S1:*** *G*Power tool outputs, comparing Ashcroft scores and micro-CT parameters (predicted % poorly aerated tissue and 70^th^ percentile) as input values for sample size planning. Both experiments (testing BLM vs drug1 and drug2) show an expected number of mice reduced by one-half when calculated using in-vivo parameters.*
