# Supplementary figures and images for: In-vivo lung fibrosis staging in a bleomycin-mouse model: a new micro-CT guided densitometric approach
Source: Sci Rep. 2020 Oct 30;10:18735. doi: 10.1038/s41598-020-71293-3 (PMC7603396; doi:10.1038/s41598-020-71293-3)

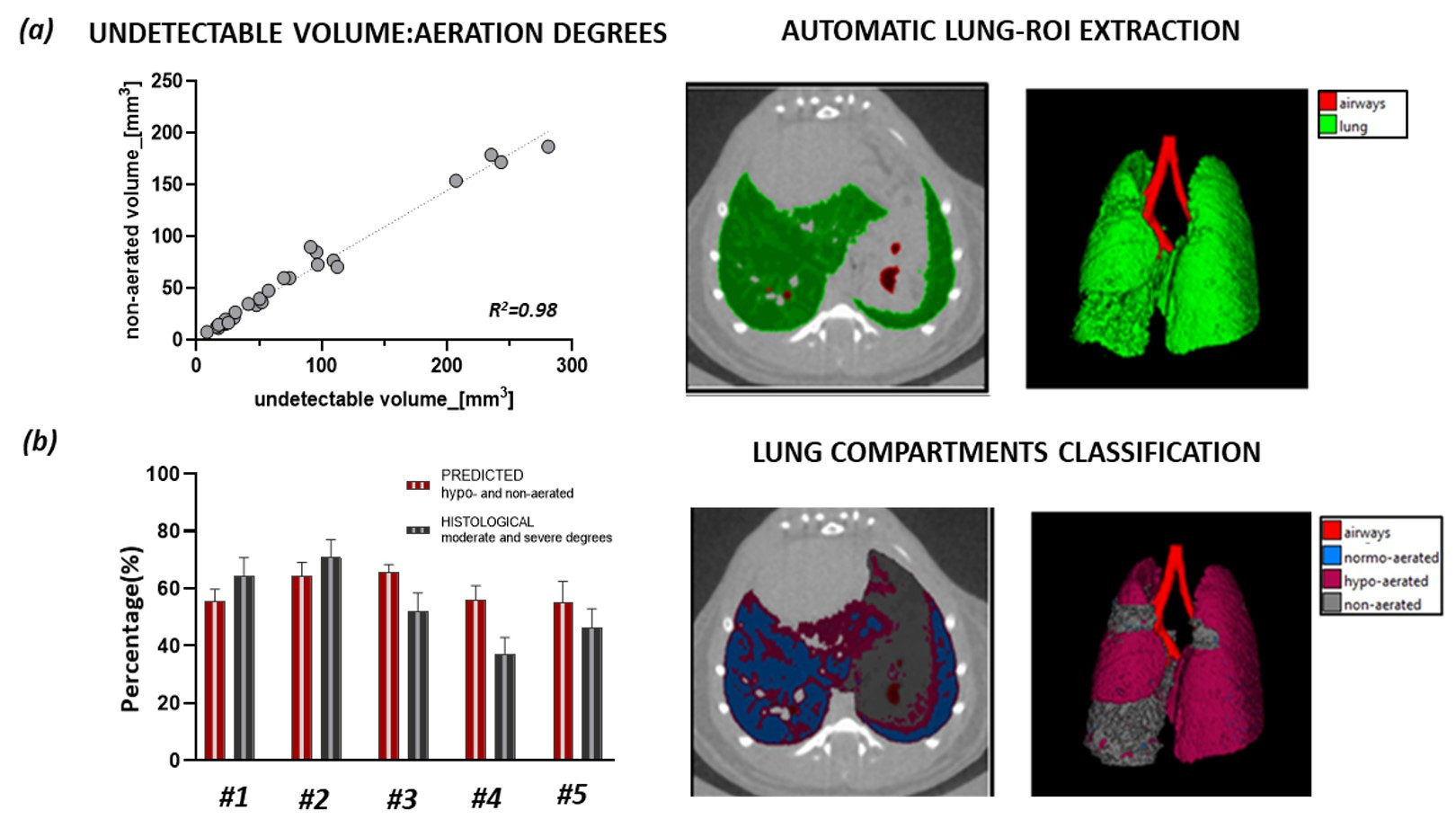

Supplement: Supplementary file 2 — Supplementary file2 [file 41598_2020_71293_MOESM2_ESM.jpg]

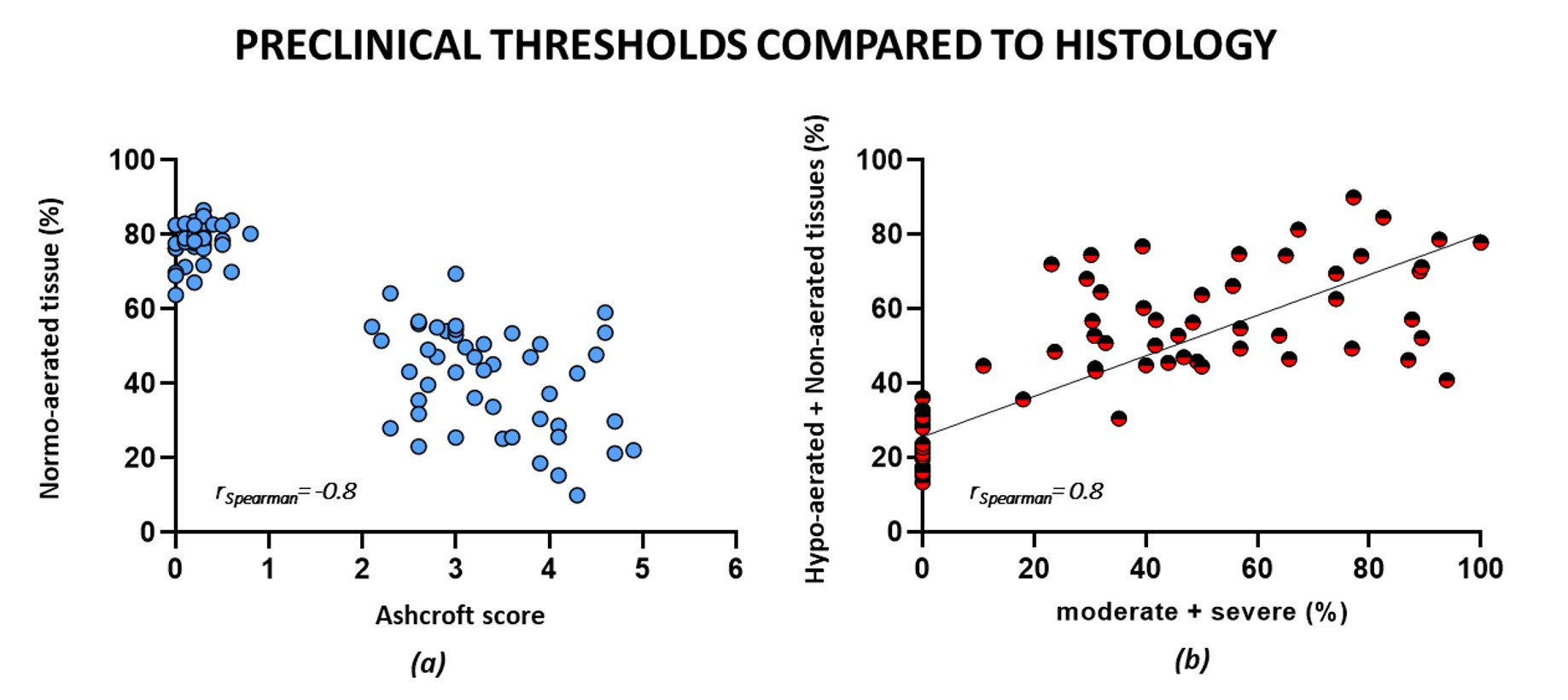

Supplement: Supplementary file 3 — Supplementary file3 [file 41598_2020_71293_MOESM3_ESM.jpg]

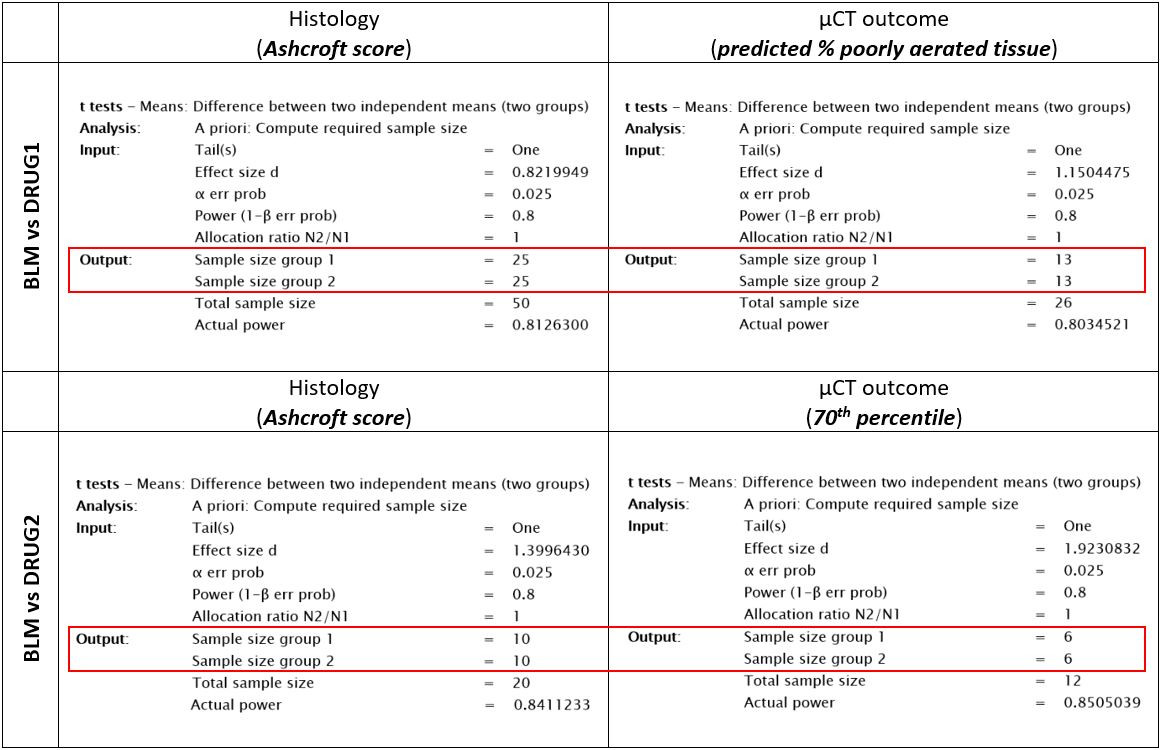

Supplement: Supplementary file 4 — Supplementary file4 [file 41598_2020_71293_MOESM4_ESM.jpg]
